# Role of the Axonal Initial Segment in Psychiatric Disorders: Function, Dysfunction, and Intervention

**DOI:** 10.3389/fpsyt.2014.00109

**Published:** 2014-08-21

**Authors:** Wei-Chun Jim Hsu, Carol Lynn Nilsson, Fernanda Laezza

**Affiliations:** ^1^Department of Biochemistry and Molecular Biology, The University of Texas Medical Branch at Galveston, Galveston, TX, USA; ^2^Graduate Program in Biochemistry and Molecular Biology, The University of Texas Medical Branch at Galveston, Galveston, TX, USA; ^3^M.D.–Ph.D. Combined Degree Program, The University of Texas Medical Branch at Galveston, Galveston, TX, USA; ^4^Department of Pharmacology and Toxicology, The University of Texas Medical Branch at Galveston, Galveston, TX, USA; ^5^Sealy Center for Molecular Medicine, The University of Texas Medical Branch at Galveston, Galveston, TX, USA; ^6^Center for Addiction Research, The University of Texas Medical Branch at Galveston, Galveston, TX, USA; ^7^Center for Biomedical Engineering, The University of Texas Medical Branch at Galveston, Galveston, TX, USA; ^8^Mitchell Center for Neurodegenerative Diseases, The University of Texas Medical Branch at Galveston, Galveston, TX, USA

**Keywords:** axonal initial segment, psychiatric disorder, neuronal excitability, neuroplasticity, signaling pathways, RDoC

## Abstract

The progress of developing effective interventions against psychiatric disorders has been limited due to a lack of understanding of the underlying cellular and functional mechanisms. Recent research findings focused on exploring novel causes of psychiatric disorders have highlighted the importance of the axonal initial segment (AIS), a highly specialized neuronal structure critical for spike initiation of the action potential. In particular, the role of voltage-gated sodium channels, and their interactions with other protein partners in a tightly regulated macromolecular complex has been emphasized as a key component in the regulation of neuronal excitability. Deficits and excesses of excitability have been linked to the pathogenesis of brain disorders. Identification of the factors and regulatory pathways involved in proper AIS function, or its disruption, can lead to the development of novel interventions that target these mechanistic interactions, increasing treatment efficacy while reducing deleterious off-target effects for psychiatric disorders.

## Disorders of the Brain

The human brain is known for its incredible complexity and heterogeneity. As such, it is susceptible to disorder and dysfunction, which in and of themselves are vastly diverse, ranging from frank physical lesions and trauma to complex changes in intracellular pathways (1). This extensive spectrum of brain disorders can generally be divided into two categories: neurologic and psychiatric. Neurologic disorders are those with a focal cause: an isolated traumatic, ischemic, neoplastic, or other insults. These include conditions such as ischemic strokes and epilepsy, and are characterized by a broadly robust understanding of the cause, if not the probable treatments. Psychiatric disorders, on the other hand, have classically been defined by the absence of an “organic lesion.” Several of the major mood disorders such as depression, schizophrenia, and bipolar disorders fall under this category (2, 3). Importantly, for much of the history of psychiatry, treatments for such disorders have relied on empirical evidence. Physicians first tried treatment that had “worked in the past,” and resorted to alternatives, also based on poorly generalizable empirical evidence, if that treatment failed.

Current widely used treatments against psychiatric disorders are fraught with mixed efficacy, poor patient tolerability, and high rates of relapse. The Sequenced Treatment Alternatives to Relieve Depression (STAR*D) study (4, 5), one of the largest studies of treatment-resistant depression to date, reported that less than half of the enrolled patients achieved remission with a level 1 treatment protocol. More troubling yet, patients who progressed through later stages suffered progressively worse remission rates, and a substantial minority remained resistant to all conventionally used antidepressant treatments. For bipolar depression with psychosis, a particularly difficult form of depression to combat, treatment remains elusive. Conventional antidepressant medications, as demonstrated in the systematic treatment enhancement program for bipolar disorder (STEP-BD) study, can actually worsen outcomes and induce shifts to manic/hypomanic states (6). Furthermore, as demonstrated in the clinical antipsychotic trials of intervention effectiveness (CATIE) study (7), even current effective frontline treatments for disorders such as schizophrenia come with a myriad of side effects. These include significant weight gain, suicidal ideation, changes in sex drive, and gynecomastia, leading to a discontinuation rate of 74% in phase 1 of patient trials (8).

These observations and other small-scale clinical trials highlight the need for new approaches to develop medications against psychiatric disorders. The difficulty of managing treatment-resistant depression, along with the severe side effects of conventional psychiatric medications, underscores a need to understand their underlying fundamental molecular and cellular mechanisms of dysregulation, to allow the development of more targeted interventions (9, 10).

## Finding Mechanisms of Brain Disorders at the Single Neuron Level

The enduring shift away from conceptualizing mental disorders as simple alterations of chemistry or receptor dysregulation, into a more global picture of combined genetic predisposition and dysregulated signaling, has identified new opportunities for early intervention (2, 3, 11). Genome-wide association studies (GWAS) looking for highly heritable genes for mental disorders, as well as monozygotic twin studies have provided researchers with sets of candidate genes that could be used as springboards for further in-depth studies (12). Advancement in biological tools, especially in the fields of high-throughput screening and next-generation sequencing, offers methods to rapidly filter thousands or even millions of candidate genes, compounds, or variants, vastly expanding the repertoire of data at researchers’ disposal. In addition, ambitious projects such as the NIH Brain Initiative and Human Connectome Project (10) aim to develop tools to generate and visualize such datasets, applying “big data” analytics to datasets far beyond those typically studied in the laboratory.

That said, these top–down initiatives stress the need to understand the mechanisms of psychiatric disorders from a bottom-up perspective. Despite the advances made in identifying heritable risks and genetic targets of psychiatric disorders, we currently lack a fundamental understanding of their pathophysiology (13). The need to transition from treatments focused on clinical symptoms (the “low-hanging fruit” of psychiatry) into treatments that address their fundamental pathophysiology is urgent (2). Therefore, we need an in-depth understanding of the neuron, its critical components, and how their dysregulation can lead to psychiatric disorders.

## Role of Large-Scale Genetic and Proteomics Data in Identifying New Targets Relevant for Psychiatric Disorders

The completion of the human genome map (14) was an important milestone in scientific history because it enabled the association of genetic variations with disease and the generation of technologies such as transcriptomics and proteomics. Recently, several GWAS investigating risk factors for a number of psychiatric disorders have identified several significantly dysregulated genes encoding proteins at the axonal initial segment (AIS) of neurons (15–18). These novel findings emphasize the need to understand the physiological function of the AIS, and the implications of AIS disorder and dysfunction. Recent advances in human genetic research are essential for the advancement of psychiatric research, but may not be sufficient to understand every aspect of complex disease spectrums (19, 20). New methods to integrate gene activity and proteomic datasets, derived from biobank samples with associated clinical metadata, promise to provide the field with powerful tools to more precisely define the molecular phenotypes of psychiatric illnesses (21). These new methods have already been applied to neurodegenerative disorders such as Alzheimer’s disease (20). Furthermore, systematic comparison between molecular and imaging data represents a new and powerful approach that is expected to define novel therapeutic targets and biomarkers of disease, and drug response (22, 23). However, applying large-scale genetic and proteomic approaches to psychiatric disorders requires bridging the knowledge gaps that exist in our understanding of the roles of AIS proteins at a single-cell level. The following sections will detail the role of the AIS in normal, as well as dysregulated, neurons.

## Molecular Composition of the AIS

**Figure 1:**
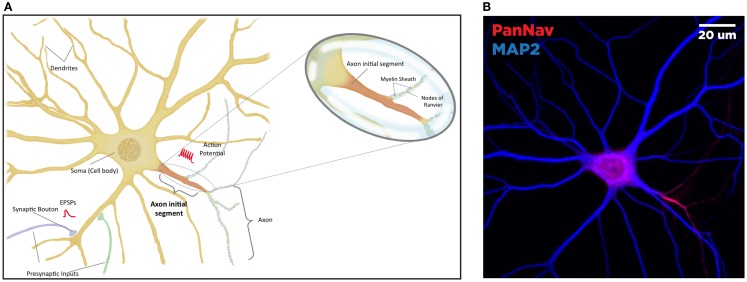
**Anatomy of the neuron**. The brain comprised functional units called neurons, which process information both through electrical and chemical signals. Communication between neurons occurs through release of chemical mediators called neurotransmitters from synaptic boutons located distally on presynaptic inputs, which diffuses through a microscopic gap (the synapse) to receptors on the receiving (postsynaptic) neuron. Neurons display both functional and spatial polarity, with multiple dendrites receiving signals and typically a single axon for sending signals, both emerging from a central soma or cell body. In contrast, communication within a neuron occurs through transient changes in membrane voltages (the action potential), which are generated primarily through movement of ions through voltage-gated sodium (Nav) and potassium (Kv) channels. These channels are highly enriched at the axonal initial segment (AIS), a protein-dense region that functions as the site of action potential initiation. Ion channels are also found in high concentrations at the nodes of Ranvier, gaps in the insulating myelin sheath surrounding the axon that allow for rapid, or salutatory propagation of action potentials. **(A)** Schematic of the neuron. Excitatory postsynaptic potential (EPSP) and action potential (AP) shown in red, at the synaptic bouton and the axon initial segment, respectively. **(B)** Confocal microscopy of a primary rat hippocampal neuron, labeled with anti-PanNav (red) and anti-MAP2 (blue) antibody visualizing axonal and somatodendritic compartments, respectively.

In neurons, the AIS serves as a nexus, integrating information received from dendrites and converting it to an electrical output (Figure 1A). This highly regulated site, proximal to the soma and delineating the beginning of the neuronal axon, is characterized primarily by a high density of voltage-gated sodium (Nav) and potassium (Kv) channels. These channels drive the initiation and propagation of the action potential (AP), and interact with several scaffolding and regulatory proteins to maintain electrical signaling (24, 25). Through the expression of specific scaffolding proteins and post-translational modification targeting, the AIS serves as a diffusion barrier to maintain the asymmetric distribution of axonal and dendritic proteins, ensuring that the neuron maintains cellular and electrical polarity (26–29). Due to this critical role in enforcing electrical signal directionality, disruptions or dysregulation of the molecular composition of the AIS is severely deleterious for proper neuronal function (30–32).

The AIS is highly enriched with several different isotypes of Nav and Kv channels, including the isoforms Nav1.1, 1.2, and 1.6 (33). Nav channels are transmembrane proteins comprising a large α subunit necessary and sufficient for Na^+^ conduction, and regulatory β subunits that can alter gating properties and localization (34). There are nine identifiable α-subunits, which are composed of four domains, each with six membrane-spanning segments (S1–S6), and multiple intracellular segments engaged in protein–protein interactions (PPI). In the AIS, these α-subunits exhibit distinct patterns of spatial and functional segregation (24, 35). The selective passage of Na^+^ into the cell is regulated via voltage-dependent switching between open, closed, and inactivated conformations. In contrast, Kv channels allow the outward flow of potassium, producing a hyperpolarizing effect; they are highly diverse, residing in 12 distinct classes and dozens of identifiable isoforms (36). Similar to Nav, Kv channels exhibit voltage-dependent conformational states that allow a regulated flux of K^+^ out of cells.

**Figure 2:**
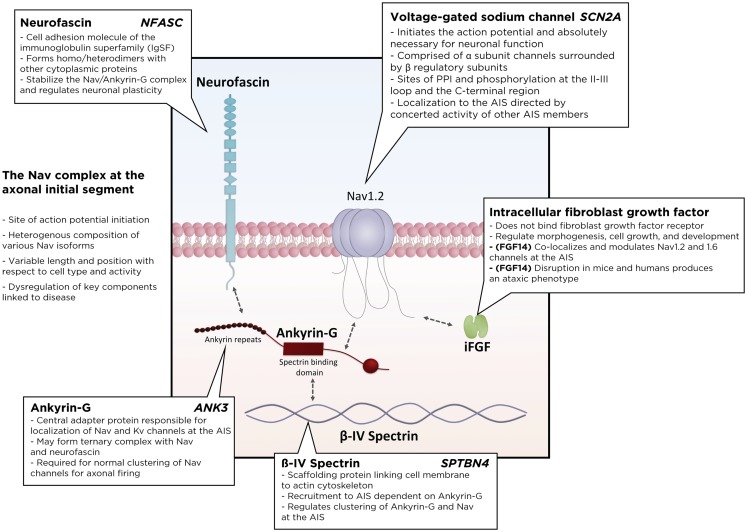
**Schematic of the Nav macromolecular complex at the axonal initial segment (AIS)**. Voltage-gated sodium channels form a tightly regulated complex with several critical regulatory and scaffolding proteins, including ankyrin-G, β4-spectrin, neurofascin, and intracellular fibroblast growth factors. PPI, protein–protein interactions.

Many accessory proteins play a critical role in regulating both the trafficking and function of Nav and Kv channels at the AIS (Figure 2). One of the key scaffolding proteins that regulates localization of Nav and Kv channels to the AIS is ankyrin-G, one of a family of proteins that associate with several ion channels as well as integral membrane proteins (37–39). Ankyrin-G interacts with a cytoplasmic loop between domains II and III on Nav channels, and C3, a C-terminal domain containing an ankyrin-G binding loop on KCNQ2/3 Kv channels. Downregulation of ankyrin-G expression through siRNA blocks the clustering of Na^+^ and K^+^ channels at the AIS (39–43). In addition, ankyrin-G recruits the critical scaffolding protein β-IV-spectrin, a process dependent on interactions between ankyrin-G and the spectrin repeat 15 (44), which stabilizes the clustering of Nav at the AIS (45) and plays an important role in the development of the nodes of Ranvier (46). Ankyrin-G also associates with one of the L1CAM family of cell adhesion molecules, neurofascin, which is alternatively spliced into several biologically relevant isoforms, including NF186, NF180, NF166, and NF155 (39). Crucially, the NF186 isoform is strongly enriched at the AIS and nodes of Ranvier, where it interacts with and stabilizes the Nav/ankyrin-G complex (47). FGF14, a member of the intracellular FGFs (iFGF; FGF11-13), is a non-secreted neuronal protein (48, 49) and an integral component of the AIS (50, 51). Through a direct interaction with the C-terminal tail of the Nav channel α subunit, FGF14 controls channel gating and Nav channel expression at the AIS. Loss of FGF14 function decreases Na^+^ currents, reduces the expression of Nav channels at the AIS, and impairs neuronal excitability, and deletion of the *fgf14* gene in rodents impairs excitability and neuroplasticity (52–54). In addition, postsynaptic density-93 (PSD-93) functions as a scaffolding protein that mediates K^+^ channel clustering at the AIS. Knockdown of PSD-93 in hippocampal neurons, as well as silencing in PSD-93^−/−^ mice, disrupts Kv1 channel clustering at the AIS (55).

Additionally, several different kinases have been described to localize or exert control at the AIS. Protein kinase casein kinase 2 (CK2), a serine–threonine kinase expressed early in neuronal development (56), is highly enriched near the ankyrin-binding motif of Nav1.2 channels, where its phosphorylation of several serine residues (S1112, S1124, and S1126) is critical for interaction with ankyrin-G (57). Knockdown of CK2 impaired its own clustering at the AIS, as well as concentrations of ankyrin-G, pIκBα, and Nav channels, suggesting a role for CK2 in axonal development (58). Calmodulin-dependent kinase (CaMKII), a serine/threonine kinase with diverse regulatory functions in ion transporter function, transcription, and cell death, is targeted to the AIS through interaction with the CaMKII-binding motif of β-IV-spectrin. A C-terminal truncation of β-IV-spectrin resulted in aberrant targeting of CaMKII, while localization of ankyrin-G and spectrin at the AIS was normal (59). Cyclin-dependent kinase (Cdk)-dependent phosphorylation of the Kv2β subunit inhibits the interaction of Kv2β with microtubule proteins. Inhibition of Cdk with the pharmacological inhibitor roscovitine, enriched Kvβ2, Kv1, and the microtubule plus end-tracking protein EB1 at the AIS (60). Additionally, glycogen synthase kinase-3 (GSK-3), a multifunctional kinase important for neuronal survival and cellular response to stress (61), establishes neuronal polarity through signaling into pathways responsible for cytoskeletal organization and microtubule stabilization (62). It has also been implicated in the development of mood disorders including bipolar disorder, depression, and schizophrenia (63, 64). In fact lithium, one of the first psychotropic drugs identified and confirmed through clinical trials to be effective against bipolar disorder, does so in part through inhibition of GSK-3 (65–67). Although this mechanism is only one of many that underlie lithium’s therapeutic potential, selective GSK-3 inhibitors, largely ATP competitors, have been reported to have an antidepressant-like effect in mice (68–71), with modulation of the upstream PI3K–Akt–GSK-3 pathway also playing a critical role (72, 73). Recently, it has been shown that inhibition of GSK-3 reduces interactions between FGF14 and Nav channels, and in hippocampal neurons, it induces redistribution of the FGF14–Nav complex, causing a reversal of axo-dendritic polarity (74). These observations suggest the existence of a GSK-3-dependent signaling pathway in the maintenance of basal neuronal polarity; a pathway that might be impaired or modified in psychiatric disorders (75).

## Functional Role of the AIS

There are several theories under consideration to explain why AIS is the site of AP initiation. Initial studies identified the AIS as a site of enrichment of Nav and Kv channels (76). However, later studies suggest that the more hyperpolarized voltage-dependence of Nav channels at the AIS better explains the low threshold of activation than channel density (35, 77, 78). Other variable properties such as gating kinetics and ion channel post-translational modification may also underlie the low initiation threshold of the AIS through alteration of open channel probabilities (79).

The heterogeneous composition of the AIS is maintained through segregation of ion channel subtypes into distinct microdomains with consequences for neuronal function. Immunofluorescence experiments with Nav specific and Pan–Nav antibodies have revealed three distinct domains of the AIS: a proximal portion of the AIS enriched in Nav1.1 and 1.2 channel subtypes, a medial portion with high levels of Kv1.2 channels, and a long distal portion enriched in lower-threshold Nav1.6 channels (35, 80). This segregation might underlie the mechanism behind the two forms of AP propagation: forward transmission through the action of Nav1.6 channels, and backpropagation through Nav1.2 channels. Confirming this hypothesis, the removal of Nav1.2 channels from the AIS region abrogated AP backpropagation (35).

The relative position of the AIS is cell type dependent and may be an important player in functional heterogeneity among various types of neurons (25, 81). Chronic depolarization of hippocampal neurons with high extracellular potassium has been observed to shift the components of the AIS, including Nav, β-IV-spectrin, neurofascin, and FGF14, distally from the soma with a corresponding decrease in firing rate (82). Furthermore, this shift was replicated via photostimulation of channelrhodopsin-2 transfected neurons and was reversible as well as frequency, interval, and Ca^2+^ dependent (82). These observations suggest that the relative position of the AIS along the axon may modulate neuronal excitability.

In summary, the diversity of AIS composition and localization may contribute to the diversity and specialization of function among different neuronal types. Disruption of AIS organization, due to injury, disease, or aging, can have profound effects for nervous system function, and may explain the pathophysiology of a variety of psychiatric disorders.

## Functional Relevance of the AIS for Psychiatric Disorders

The AIS is undoubtedly a crucial hub for neuronal function. Thus, dysfunction or dysregulation of AIS components predicts important consequences for neuronal function and has been identified as a causative component for many psychiatric and neurological disorders (Table 1).

**Table 1:** **A selection of human disorders associated with dysregulation of key AIS components**.

Class	Gene	Associated disorders
**Voltage-gated sodium channels**	***SCN1A***	Dravet syndrome (severe myoclonic epilepsy of infancy) (83, 86)
		Sporadic autism and familial autism (92)
		Autism spectrum disorders (93)
		Familial hemiplegic migraine (95)
		Mesial temporal sclerosis (96)
	***SCN1B***	Possibly linked to Dravet syndrome (86)
	***SCN2A***	Ohtahara syndrome (infantile epileptic encephalopathy) (89)
		Autism spectrum disorders (92)
**Ankyrins**	***ANK3***	Bipolar disorder (16, 97, 98)
		Schizophrenia (99, 101–103)
		Post-traumatic stress disorder (99)
		Late-onset Alzheimer’s disease (104)
**Spectrins**	**All**	Aging and Alzheimer’s (108, 109)
	***SPTAN1***	West syndrome (infantile spasm) (112)
	***SPTBN2***	Spinocerebellar ataxia type 5 (160)
	***SPTBN4***	Combined spherocytosis and autism (106)
		Auditory and motor neuropathies (107)
**L1 family IgSFs**	***NFASC***	Multiple sclerosis (116–118)
		Central and peripheral demyelination disorder (116–118)
	***CHL1***	Schizophrenia (120)
		Mental retardation (122–124)
**Intracellular FGFs**	***FGF14***	Spinocerebellar ataxia type 27 (50, 130, 131)
		Paroxysmal dystonia (137)
		Cognitive impairment (138)
		Major depressive disorder (133)

Mutations of Nav have been linked with various neurological and psychiatric disorders. Dravet Syndrome is a form of severe myoclonic childhood-onset encephalopathy caused by *de novo* heterozygous mutations in Nav1.1 in which numerous epileptic phenotypes are seen including febrile, absence, myoclonic, and atonic seizures (83). A mouse model with a C-terminal truncation of Nav1.1 reproduced severe ataxia, seizures, and premature death (84), and heterozygotes exhibited substantially reduced sodium current density in GABAergic inhibitory neurons (85). Thus, decreased GABAergic inhibition may result in increased seizurogenic currents, resulting in the severe seizures seen in myoclonic childhood-onset encephalopathy. In addition, generalized epilepsy with febrile seizures plus (GEFS+), a large set of autosomal dominant disorders that includes Dravet, encompass various mutations in *SCN1A* and *SCN1B* as well as genes encoding GABA receptors and calcium channels. Unlike patients with Dravet, who predominantly display truncation and missense mutations in the *SCN1A* gene, patients with GEFS+ display a much more variable clinical presentation (86). Additionally, missense Nav1.1 (87, 88), Nav1.2 (89), and Nav1.6 (90) mutations have been implicated in various forms of genetic epilepsies, and in fact Nav1.1 testing for genetic epilepsies is used in the clinic (91). Dysfunction of Nav has also been linked to autism. Early studies identified 38 significant SNPs in *SCN1A, SCN2A*, and *SCN3A* in a cohort of 117 families with a history of genetic autism (92). More recently, genome sequencing of individuals with autism spectrum disorder have revealed multiple independent *de novo* SNP variants of Nav1.2 that are highly associated (93) and another group has identified a highly interconnected protein interaction network of *de novo* mutations, including disruptions in Nav1.1 in sporadic autism (94). Also, mutations of Nav1.1 have been observed in patients with familial hemiplegic migraine and epileptic phenotypes (95), and may be associated with hippocampal sclerosis (96). Due to the range of polymorphisms identified for Nav channels, further investigation into their relevance to the development of psychiatric disorders is needed.

In addition to Nav, dysfunction of the scaffolding protein ankyrin-G has been implicated in a number of psychiatric disorders. Ankyrin-G has been identified in GWAS as a potential risk gene for bipolar disorder with significantly associated SNPs in ANK3 as well as other bipolar disorder candidate genes (16, 97), including one that maps to a non-conservative amino acid change (98). Supporting the association of ANK3 with stress-related behaviors, a recent study has linked ANK3 SNPs with both post-traumatic stress disorder and externalizing (a measure identifying adult anti-sociality and substance abuse) in a cohort of military veterans (99). In addition, RNAi downregulation of ANK3 in the dentate gyrus of the mouse hippocampus decreased anxiety-related behaviors and increased activity during the light phase. These behaviors are consistent with increased stress and mood episodes in BD (100). ANK3 polymorphisms have also been associated with schizophrenia (101). Schizophrenic patients with ANK3 polymorphisms exhibit reduced ankyrin-G density in the superficial dorsolateral prefrontal cortex and superior temporal gyrus compared with normal subjects. This has important consequences for pyramidal neuron connectivity, and speech and emotional processing (102, 103). Additionally, genome-wide studies support an association between ANK3 and late-onset Alzheimer’s disease in the LOD-1 linkage region (104). In fact, vaccination with ankyrin-G in arcAβ transgenic mice (a classic Alzheimer’s model) reduced β-amyloid pathology in the brain and increased soluble levels of Aβ. In addition, hippocampal arcAβ neurons treated with monoclonal anti-AnkG antibodies displayed a reduction in the loss of dendritic spines (105).

Given the tight association between ankyrin-G and β-IV-spectrin, dysregulation of spectrin expression or function can also be expected to contribute to several psychiatric disorders. Though research is currently lacking, the *de novo* microdeletion of a gene region containing β-IV-spectrin was observed in a patient with a combined autism/spherocytosis phenotype (106). In mice, truncation of β-IV-spectrin causes auditory as well as motor neuropathies, resulting in the autosomal recessive *quivering* phenotype (107). More broadly, spectrin catabolism through calpain-mediated cleavage, seen clinically in aged and patients with Alzheimer’s disease (108, 109), has been associated with stimulation of *N*-methyl-d-aspartate (NMDA) receptors. This results in elevation of intracellular calcium levels as well as increased levels of spectrin breakdown products. These contribute to neuronal degeneration through disruption of intracellular transport and calcium toxicity (110, 111). Both α- and β-spectrin disruptions have been implicated in nervous system dysfunction. Heterozygous mutations in β-III-spectrin produce a slowly progressive, autosomal dominant spinocerebellar ataxia; a condition that exhibits the degeneration of cerebellar Purkinje cells and associated morphological abnormalities observed in a mouse knockout of β-III-spectrin. Mutations in α-spectrins are associated with West Syndrome, an epilepsy disorder characterized by infantile spasms, atrophy of the corpus callosum and cerebellar vermis, and developmental regression (112). Further research is needed to determine the pathophysiology behind these disorders and identify the roles that non-β-IV spectrins may play in regulation of the AIS.

In addition to Nav and spectrin, ankyrin-G also interacts with L1CAM cell adhesion molecules. Isoforms of neurofascin and NrCAM, important members of the L1CAM family, colocalize with ankyrin-G at the AIS (113). Neurofascin plays a critical role in stabilizing complexes of Nav channels and ankyrin-G (47, 114), so defects in neurofascin will adversely affect neuronal function. Knockdown of neurofascin in adult rat brain impairs GABAergic expression at synapses localized at the AIS, which suggests its function in modulating axo-axonal innervation (114). Furthermore, inactivation of neuronal neurofascin (NF186) in adult mice results in AIS degeneration, Purkinje cell dysfunction, and impaired motor learning (115). In human patients, axonal injury characteristic of multiple sclerosis (MS) and other demyelination disorders is associated with increased concentrations of autoantibodies against both NF155 and NF186, which are present in inflammation involving complement deposit, microglial activation, and ultimately axonal injury (116–118). More broadly, defects in the L1 gene family, which encodes various cell adhesion molecules including neurofascin (119), have been observed in a variety of neurological and psychiatric disorders. SNPs in CHL1 are associated with an increased risk of schizophrenia (120) Postmortem analysis of hippocampus and amygdala from schizophrenic patients revealed decreased polysialylated neural cell adhesion molecule (PSA-NCAM) expression in parallel with decreased GAD67 and increased VGLUT1 levels in white matter (121). In addition, patients with chromosome 3p aberrations (which include several brain-expressed genes including *contactin* and *CHL1*) present clinically with mental retardation. A subset of those also exhibit physical features such as skeletal defects and facial dysmorphism (122–124). Supporting the role of *CHL1* in mental retardation, *Chl1*-heterozygote and homozygote mice exhibit a gene dosing effect. *Chl1*^−/−^ mice display altered exploratory patterns in the Morris water maze test, increased sociality, and reduced aggression (125), suggesting that processes involving cognition and spatial memory are disrupted. In addition, phosphorylation of L1 increases its association with doublecortin, which is important for neuronal migration (126), and is downstream of MAP kinase pathways (127). This suggests that ERK-dependent signaling may promote neurite growth through downregulation of L1 and ankyrin binding (128, 129). These observations highlight the diversity of the L1 gene family, and the need for more research into how other components of the L1 family interact at the level of the AIS.

Mutations of the intracellular fibroblast growth factor FGF14 have been associated with hereditary spinocerebellar ataxia (130, 131), a debilitating childhood-onset condition characterized by postural tremor, slowly progressive ataxia, and cognitive deficits. Specifically in SCA27, the FGF14^F145S^ mutation decreased Nav currents and reduced neuronal excitability in hippocampal neurons by disrupting the FGF14:Nav interaction by a dominant negative inhibition of the FGF14 wild-type form (50, 131). Further analysis of the P149Q polymorphism on a closely related homolog, FGF12, revealed a loss of pairwise specificity with the C-terminal end of Nav1.1 and subsequent loss of Na^+^ channel function modulation, suggesting that mutations in this region may dysregulate neuronal excitability (132). Additionally, a genome-wide study performed on a Dutch major depressive disorder cohort identified SNPs covering seven candidate genes including FGF14 (18). Previous studies have implicated extracellular FGF activity in stress and major depressive disorder (133). In fact, increases in hippocampal FGF activity may be one of the mechanisms of antidepressants such as fluoxetine, desipramine, and mianserin (134–136). In mice, targeted disruption of FGF14 produces severe ataxia, paroxysmal dystonia, and cognitive impairment (137, 138), with neurons that exhibit severe impairments in synaptic plasticity (53) and neuronal excitability (52). Significantly, the assembly and trafficking of FGF14 itself is controlled via a GSK-3-dependent signaling pathway (74) that may critically regulate excitability through PPI at the level of the Nav complex. Additionally, the distribution of FGF14 is Nav channel dependent. Deletion of the Nav1.6 α subunit in mouse Purkinje neurons markedly increases FGF14 levels in the AIS, in parallel with increased expression of Nav1.1 and β-IV-spectrin (139). As a result, through interactions with macromolecular complexes at the AIS, FGF14 plays an important and underappreciated role in the regulation of Na + channel activity.

## AIS Targeting as a Pharmacological Strategy

Given the association between defects at the level of AIS and subsequent impairment of neuronal function that leads to the development of several important neurological and mental disorders, interventions directed at restoring AIS function may be more effective. Targeting AIS function would allow for the development of highly specific, efficacious compounds with significantly reduced off-target effects and enhanced clinical outcomes (13, 140, 141). Given the importance of the molecular composition of the AIS to disease, selective targeting of PPI at the AIS may deliver a new generation of treatments against psychiatric disorders.

### Improving diversity through protein–protein interactions

Inhibition of PPI remains a novel and increasingly attractive area of drug design. Although nascent, it has the potential to tremendously expand the repertoire of targets available for the pharmaceutical industry (142, 143). Through the engineering of small molecules or peptidomimetics (144, 145) that interact with and disrupt specific protein–protein interfaces, the effects of the inhibitor on the signaling network of the cell would be restricted from *n* edges (*n* = number of potential PPI between the targeted protein and the *entire* proteome) to a single edge. This would vastly increase the potential specificity without diminishing effectiveness. In addition, selective targeting of protein surfaces prevents global disruption of protein expression levels, which may upregulate alternative and compensatory pathways that reverse treatment effects and replace critical “disease-promoting” factors, leading to relapse (144). In the context of dysregulation at the AIS, this targeted multi-protein approach has the potential to restore function as a consequence of a specific deficiency while having a minimal impact on other properly functioning pathways.

Approaches to discover PPI of interest are cooperative, and include yeast two-hybrid systems, RNAi interference, protein fragment complementation, mass spectrometry, and direct co-immunoprecipitation. Large-scale studies using high-throughput technologies in combination with algorithmic approaches have identified hundreds of thousands of protein interactions based on structural evidence and biochemical assays (146, 147). However, the design of small molecules to exploit these PPI presents an entirely new set of challenges, especially if the PPI are part of ion channel complexes, as is the case at the AIS (143). Many studies use computational modeling in conjunction with HTS approaches to screen databases of “drug-like” compounds for molecules that bind to “hot-spots”; regions within the protein near the protein–protein interface. Alternative approaches could include the study of allosteric PPI modulators, compounds that bind at a site distant from the interaction interface that induce conformation changes which alter the PPI, as well as fragment-based approaches that involve searching two separate compounds joined by a linker to better probe chemical space (148, 149). However, the difficulty of translating biochemical assays to actual human therapies manifests as concerns in stability, pharmacokinetics/bioavailability, and toxicity, and poses a substantial barrier toward effective therapies that target the PPI (144).

Nonetheless, therapeutic targeting of PPI is an intensive area of interest in oncology, with preliminary studies in brain disorders. A number of BCL-2/BH3 PPI inhibitors such as GX15-070 and AT-101 are used in early clinical trials for a variety of solid tumors, including lung carcinoma and GIST, with more expectation (150, 151). Utilizing a multiple ligand simultaneous docking (MLSD) model, a group has identified raloxifene and bazedoxifene as potent inhibitors of the IL-6/GP130 interface, a critical step in the STAT3-mediated pathway of cancer progression (152). In the context of cerebral ischemia and neuropathic pain, GABAB receptors are often excessively upregulated (153, 154), and small peptides interfering with GABA-interacting proteins such as 14–3–3 may selectively restore normal GABA function without affecting the GABA receptors not implicated in disease (155, 156). Disruption of metabotropic glutamate receptor (mGluR7a) interaction with proteins interacting with kinase 1 (PICK1) has been shown to induce the absence of seizures in rodents, suggesting that small molecules that stabilize this interaction could be potential therapeutics against epilepsy (157). Additionally, interaction of the serotonin 5HT2C receptor with multiple PDZ (MPDZ), a gene found to be dysregulated in physiological drug dependence (158), has been explored as a potential avenue of research in addiction phenotypes, and small molecule inhibitors of the MPDZ–5HT2C interaction have been developed (159).

These early successes highlight the potential for harnessing PPI at the AIS as a novel approach toward better interventions against psychiatric disorders. Through the engineering of novel therapeutics that target dysregulation at the interaction level, versus the protein level, there is much potential for greater specificity and diversity in the design of useful psychiatric treatments.

## Conclusion

Despite the immense challenges in treating patients with psychiatric disorders, next-generation treatments based on rational understanding of the underlying mechanisms of dysregulation hold much promise. Early, top-down high-throughput studies to identify focal points of dysfunction, as well as bottom-up reductionist studies of the core components of neurons, including the AIS, will invariably lead to a better understanding of the pathophysiology behind psychiatric disorders. Whether this new-found understanding leads to concrete treatments for psychiatric disorders – the middle-aged mom of two suffering from yet another relapse of depression; the hard-working, successful executive crippled by a decade-long struggle with schizophrenia; the college freshman terrified by yet another manic episode – remains to be seen, but the urgent need for more effective, less deleterious treatments is undeniable.

## Methods

### Neuronal cultures and confocal microscopy

Primary hippocampal cultures were obtained from E18 rat embryos using Banker’s method and processed for immunofluorescence as previously described (74). The image displayed in Figure 1B was obtained using rabbit anti-PanNav and chicken anti-microtubule-associated protein 2 (MAP2) primary antibodies followed by incubation with appropriate Alexa-conjugated secondary antibodies for immunofluorescence visualization. Confocal images were obtained at a magnification of 63× with a Zeiss LSM-510 Meta confocal microscope.

## Conflict of Interest Statement

The authors declare that the research was conducted in the absence of any commercial or financial relationships that could be construed as a potential conflict of interest.
